# Physical Activity Behavior from a Transdisciplinary Biopsychosocial Perspective: a Scoping Review

**DOI:** 10.1186/s40798-020-00279-2

**Published:** 2020-10-17

**Authors:** Jannika M. John, Vanessa Haug, Ansgar Thiel

**Affiliations:** 1 Institute of Sports Science, Eberhard Karls University Tübingen, Wilhelmstraße 124, 72074 Tübingen, Germany; 2 Interfaculty Research Institute for Sport and Physical Activity, Eberhard Karls University Tübingen, Tübingen, Germany

**Keywords:** Biopsychosocial, Physical activity, Socio-ecological, Framework, Approach, Scoping review

## Abstract

**Background:**

Physical activity behavior is a complex and multidimensional phenomenon. For its analysis, transdisciplinary biopsychosocial approaches yield great potential. In health research, the biopsychosocial model has experienced a renaissance. Researchers have tried to grasp the complex interplay of biological, psychological, and social factors. With this scoping review, we aimed to examine how the ‘biopsychosocial’ has been conceptualized in scientific work related to physical activity behavior.

**Methods:**

The scoping review was informed by the PRISMA guidelines for scoping reviews (PRISMA-ScR). A systematic literature search was conducted in Web of Science, SportDiscus, PsycArticles, PsycInfo, and PubMed. Only articles published in peer-reviewed journals that contained all three components of a biopsychosocial approach (e.g., bio/physio/genetic, psycho/mental, and socio/cultural/environmental) were included. We only included articles in our narrative synthesis that integrated physical activity behavior into a biopsychosocial model, or investigated or described physical activity behavior on the basis of such a model.

**Results:**

Thirteen studies met the inclusion criteria; eight articles pursued a biopsychosocial approach in the tradition of Engel, five employed a socio-ecological approach. The models in the analyzed articles referred to either correlates of physical activity behavior, or the influence of physical activity on health or aging. Only a minority of the articles, however, referred to interactions between biological, psychological, and social factors.

**Conclusions:**

The included articles were quite heterogeneous in their approach to physical activity from a biopsychosocial perspective. The included articles illustrate that the adoption of a biopsychosocial perspective may assist to capture and understand the complex phenomenon of physical activity behavior and might inform future transdisciplinary physical activity research.

## Key Points

- The complex and dynamic nature of physical activity behavior requires a transdisciplinary perspective integrating the interplay of biological, psychological, and social factors.
- Existing literature does not give clear answers on how biological, psychological, and social factors are interwoven with regard to the uptake, maintenance, and effectiveness of physical activity behavior.
- The present scoping review not only shows the potential of looking at physical activity behavior from a biopsychosocial perspective but also highlights the need for further empirical and conceptual research in this area.

## Introduction

There is a broad agreement among researchers that physical activity (PA) is a multidimensional and complex phenomenon. Its analysis therefore requires complex, biopsychosocial approaches [1] that can enable a “better understanding of those characteristics of individuals that are important in terms of the uptake, acceptance and maintenance of physical activity” [2]. Biopsychosocial analyses in the context of health and illness were particularly influential in the 1970s and 1980s. In the following decades, health research more and more shifted to mono-disciplinary studies. However, in recent years, the biopsychosocial model has experienced a veritable renaissance in the field of health and illness research [3]. This development could be the result of a demand, spread globally by the WHO, that health should be analyzed from a holistic perspective [4]. Hence, many studies on health and illness have tried to grasp the interplay of biological, psychological, and social factors [5].

In research on PA behavior, multidisciplinary perspectives are almost “state of the art.” However, this does not necessarily mean that the studies are based on a holistic perspective. In order to examine whether the “biopsychosocial” has also become a standard in PA research, we are therefore particularly interested in the question, to which extent research in the area of PA claims to follow a biopsychosocial paradigm. Hence, the present scoping review aims to answer the question, which theoretical and conceptual frameworks exist for the analysis of PA behavior from a biopsychosocial perspective.

### Biopsychosocial Models of Health and Illness

In general, the biopsychosocial is understood as a holistic perspective on a variety of phenomena [6]. Due to the complexity of most phenomena, the single components are therefore not considered as isolated entities, but their dimensional interactions characterize the phenomenon under question. In the context of health and illness, most definitions of the “biopsychosocial” refer to Engel’s biopsychosocial model for the explanation of health and illness [7].

This holistic biopsychosocial perspective emerged as a response to the recurring criticism of the dualistic view on body and mind and reductionism [6]. In the biopsychosocial model of health and illness, the single components at the individual system level (i.e., biological, psychological, and social) are not considered independently of each other, but the main focus is on their interactions [8]. It is precisely these interactions that stand in contrast to a purely biomedical approach where disturbances or functional constraints would only be considered at the respective system level without paying attention to the levels above or below. In contrast, health and illness in the biopsychosocial disease model are always dependent on the functionality of all individual levels and the handling of disturbances at each level. The strength of such an approach is that health and illness are not conceptualized as fixed phenomena, but as dynamic events based on the interactions between the different system levels.

### Physical Activity From a Biopsychosocial Perspective

Such a systemic and dynamic perspective also holds potential for conceptualizing PA, which is also a highly complex, dynamic, and multidimensional behavioral phenomenon [9, 10]. In recent years, the number of multidisciplinary perspectives on PA has continually increased. Today, there is a broad consensus that PA has a high potential for promoting public health due to its various positive biopsychosocial effects. From a biological perspective, many positive effects of PA, such as the prevention of diseases of the musculoskeletal system [11] and the cardiovascular system [12], are considered evidence-based. It is also widely accepted that PA has psychological effects, such as the reduction of symptoms of depression or increasing self-esteem in children and adults [12]. Social benefits of PA, such as the integration of people with a migration background, the ability to work in a team, or social interaction with others [13], are also often described, although the degree of evidence is rather low compared to the other dimensions. Taken together, the effects of PA can be described from a biopsychosocial perspective. This also applies to the correlates of PA behavior. In this regard, PA behavior is influenced by biological factors, such as genetics [2, 14], the individual state of health, psychological aspects such as motivation or affect, or social factors such as social support from significant others [12, 15].

However, existing literature does not give clear answers on how the biological, psychological, and social factors are interwoven with regard to the uptake, maintenance, and effectiveness of PA behavior. In order to get a clearer picture of what we know and do not know about the biopsychosocial analysis of PA behavior, we aim to review theoretical approaches that try to explain the correlates and effects of PA behavior from a transdisciplinary biopsychosocial perspective and integrate the interactions between the different components in their models [16, 17].

On this basis, this scoping review attempts to (a) provide an overview of theoretical and conceptual biopsychosocial frameworks related to PA behavior, (b) critically discuss how the biopsychosocial perspective is applied to research in the field of PA behavior, (c) identify key characteristics of research conducted from a biopsychosocial perspective, and (d) provide implications for future transdisciplinary PA research in the context of health promotion, disease prevention, and rehabilitation and treatment.

## Methods

The method for this scoping review was informed by the preferred reporting items for systematic reviews and meta-analyses extension for scoping reviews (PRISMA-ScR) guidelines [18]. We chose a scoping review approach because it is particularly suitable for determining the scope of a body of literature on a rather broad topic [19]. With regard to our research question, a scoping review approach allows us to investigate how the “biopsychosocial” has been conceptualized in the existing literature on PA behavior, to assess which biological, psychological, and social factors have been considered in research conducted from a biopsychosocial perspective, and to identify knowledge gaps in an attempt to provide implications for future transdisciplinary work in the field of PA research.

### Literature Search

We used the databases Web of Science, SportDiscus, PsycArticles, PsycInfo, and PubMed for the search. Additional references were identified through screening of reference sections of eligible studies. No publication date limitation was set. The search was deliberately not limited to a publication period or the type of publication in order to generate a complete overview of the available literature.

The last search was carried out on 24 June 2020, using the following search strategy with English and German search terms for all databases: (biopsychosocial OR “bio-psycho-social” OR transdisciplinary OR biopsychosozial OR transdisziplinär) AND (“physical activity” OR “körperliche Aktivität”) AND (model OR framework OR approach OR perspective OR Modell OR Ansatz OR Perspektive). For the databases Web of Science and PubMed the search setting “All Fields” was used, for the other databases the default settings were used.

Before selecting the search strategy described above, some search strategy revisions were made as part of the research process, staying true to the scoping approach of the present review [20]. In our initial search, we only searched for the term “biopsychosocial” and not for the term “transdisciplinary.” However, this resulted in a rather low number of hits. Furthermore, when screening the respective texts, we noticed that the term “transdisciplinary” was often used interchangeably with the term “biopsychosocial.” Hence, we adapted our search strategy accordingly and included “transdisciplinary” in our search strategy. We also attempted to add the search term “interdisciplinary.” However, this generated too many hits, which is why we decided to abstain from including this term in our search strategy.

In this review, we aimed to focus on biopsychosocial approaches to PA behavior in daily life. Our analysis concentrates on PA as a generic form of bodily movement integrated into one’s normal daily routine [21]. We have explicitly chosen to exclude sport and exercise from our search and analysis since both denote activities that take place in specifically structured settings with different aims. Sport as a distinct form of PA is characterized by an explicit focus on competition and performance. Exercise is practice with the aim of improving a specific ability or skill [22]. Hence, sport and exercise participation may also be influenced by different biological, psychological, social, and environmental factors and likely lead to different effects on the biological, psychological, and social level, which, as a consequence, would impede a synthesis of biopsychosocial approaches in the context of PA behavior from a more universal perspective. Additionally, the sole focus on lifestyle PA reflects the recent paradigm shift in the health literature from exercise promotion to a combination of PA promotion and reduction of sedentary time [23].

### Screening Process and Inclusion and Exclusion Criteria

Inclusion and exclusion criteria for each stage of the screening process (i.e., title, abstract, and full-text screening) were not predetermined but developed iteratively. Criteria were further refined and adapted as knowledge grew, which is in line with the notion of a scoping review [20].

Papers were included if they (i) focused on biological, psychological, and social factors influencing engagement in PA; (ii) examined biological, psychological, and social effects of PA behavior; (iii) were published in peer-reviewed journals; (iv) were published in English or German; and (v) were available as full-texts.

Papers were excluded if (i) they did not contain *all* three components of a biopsychosocial approach (e.g., bio/physio/genetic, psycho/mental, and socio/cultural/environmental); (ii) they solely focused on exercise and sports in organized and structured settings (i.e., papers examining determinants of sport participation or effects of exercise interventions).

Since we aimed to examine how the “biopsychosocial” is conceptualized in PA research, we did not use any restrictions related to the term “biopsychosocial.” As long as papers referred to a model that integrated biological, psychological, and social aspects in the context of PA behavior, they were included in the present scoping review, even if the original authors did not explicitly use the term “biopsychosocial” in their work.

**Fig. 1:**
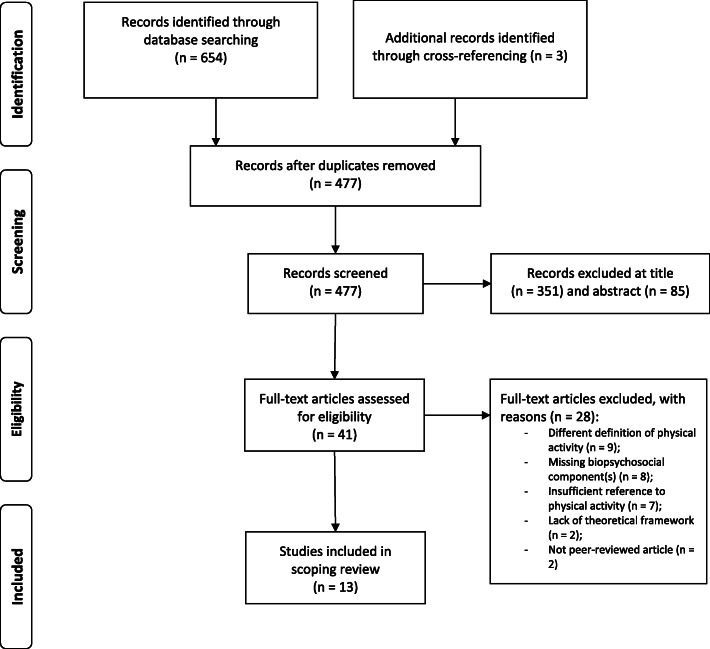
PRISMA flow diagram

At each stage of the screening process, articles that did not meet the inclusion criteria were excluded. Reasons for exclusion of full-texts are depicted in Fig. 1. The first and second author screened all titles and abstracts independently. To assess inter-rater reliability during the screening process, we calculated the kappa statistic [24]. When there were disagreements about the eligibility of particular articles, agreement was reached through a process of constructive debate between all authors.

### Study Synthesis

Extracted data included the article type, meaning whether they were empirical papers or non-empirical work (i.e., literature reviews, commentaries, theoretical work), the article’s overarching objective, and the study’s underlying theoretical model or approach. Thereby, we extracted the model’s components and identified which specific variables the original study authors operationalized among the respective components, how PA was integrated into the model, and how the nature of the relationship between the model components was depicted (see Table 1). We narratively synthesized our findings.

**Table 1:** Summary of included articles

Authors (year) [citation no.]	Research objective	Theoretical model or approach	Model’s components	Integration of PA	Relationships or interactions between components
**Empirical articles**					
Flannery et al. (2019) [25]	Examining the link between social, biological, behavioral, and psychological factors and level of PA in healthy pregnant women.	Biopsychosocial model	*Biological:* Gravidity, BMI *Behavioral:* Smoking, alcohol, folate intake, fruit and vegetables, fish *Psychological:* Anxiety, stress, depression, response to pregnancy *Social:* Age, ethnicity, marital status, employment status, accommodation, socioeconomic status, maternity service	(Possible) determinants	Unidirectional influence of the components on PA levels
Hearst et al. (2012) [26]	Examining factors that predict PA in children and adolescents between 10 and 16 years	Socio-ecological model	*Intrapersonal:* Self-efficacy, PA enjoyment, barriers to PA *Behavioral:* Screen time; sports team participation *Social:* Parent and peer support *Physical environment:* Home PA environment, neighborhood safety, walkability *Individual-level measure* (*covariates*)*:* Pubertal status, age, sex, race, weight, height, percent body fat, demographic and socioeconomic status	(Possible) determinants	Unidirectional influence of the respective components on PA
Lämmle et al. (2013) [27]	Examining the association between distal and proximal factors that influence PA, sedentary behavior, and eating behavior and relationship to health in children and adolescents between 4 and 17 years	Biopsychosocial model	*Distal* (*environmental*)*:* Socio-economic status, rural-urban differences, immigration background *Proximal I* (*interpersonal*)*:* PA of relatives and peers *Proximal II* (*intrapersonal*)*:* Motivation, psychopathological problems, quality of life *Behavioral*: PA, eating patterns, sedentary behavior *Objective health and physical fitness:* BMI, body fat, blood pressure, cholesterol *Health and health complaints:* Pain, psychosomatic and physical complaints, subjective health	(Possible) effects and determinants	Interrelationships between components
McNeil et al. (2006) [28]	Examining the relationship between individual factors and factors of the social and physical environment on PA within a group of Afro Americans and Caucasian Americans	Socio-ecological model	*Individual:* Self-efficacy, motivation *Social environmental:* Social support *Physical environmental:* Neighborhood quality, access to facilities *Sociodemographic:* Age, race, ethnicity, sex, household income, education	(Possible) determinants	Integration of different intensities of PA (unidirectional relationships); Relationships between components (but not bidirectional)
Meisner et al. (2010) [29]	Examining the relationship between PA and the three components of successful aging within a group of 60 years of age and above	Biopsychosocial model (after Rowe and Kahn [30, 31])	*Low probability of disease or disease-related disability:* Presence of chronic conditions (respiratory diseases, inflammatory diseases, inflammatory diseases, cardiovascular diseases, metabolic and related diseases, cancers, incontinence, back problems) *High cognitive and physical functional capacity:* Assistance with instrumental and general activities of daily living *Active social engagement with life*: Time spent in sedentary activities, sense of belonging to the local community, involvement in voluntary social organizations *Covariates:* Sex, age, total household income	(Possible) effects	Unidirectional influence of PA on components of successful aging
van Roekel et al. (2015) [32]	Examining the relationship between low and moderate-to-vigorous PA and the health-relevant quality of life in former colorectal cancer patients	Biopsychosocial model (based on International Classification of Functioning, Disability, and Health)	*Cancer-specific quality of life:* Global health; physical, role and social functioning; self-reported fatigue; anxiety and depression; disability *Other factors:* Sociodemographic characteristics (sex, age, education level, smoking status), BMI, presence of comorbidities, clinical characteristics	(Possible) effects and determinants	Integration of PA into the model (with bidirectional relationships); Interrelationships between components
**Non-empirical articles**					
Collins et al. (2011) [33]	Description of a multidimensional approach for lifelong sport participation and PA using a critical perspective on key theories	Biopsychosocial model of participation in PA	*Biological:* Biological maturation, readiness, hormonal change *Psychological*: Psychological development, pressure *Social*: Transitions, access, peer, social expectations	(Possible) determinants	Unidirectional influence of components on PA; Possible interactions between components over the life course
Kanavaki et al. (2017) [34]	Systematic review of barriers and facilitators to participation in PA in adults with gon- or coxarthrosis	Biopsychosocial model after Engel [35]	*Physical health*: Pain, physical capacity, age, physical fitness *Intrapersonal/psychological:* Experience and beliefs about PA, behavioral regulation and attitude, emotions *Social environment*: Health professional, social support *Physical environment:* Cost, accessibility, temperature, safety issues	(Possible) determinants	Unidirectional influence of the respective components on PA; Reference to interrelationships between components
Kanning and Schlicht (2008) [36]	Description of a biopsychosocial model for successful aging and its effects on subjective well-being	Biopsychosocial model of successful aging	*Personal disposition:* Physiological constitution/genotype, personality, socialization/sports-biography, socio-economic status *Social-structural constraints:* Stereotypes, behavior setting, offers/ facilities *Psychological:* Cognition, emotion, goals, need satisfaction, subjective well-being	(Possible) effects	Unidirectional influence of PA on subjective well-being
King and King (2010) [37]	Discussion of advantages of a healthy lifestyle, and current problems and challenges and their significance for science, politics, and practice	Socio-ecological model	*Personal:* Sex, age, genes, beliefs, enjoyment of PA, motivation, health status, function, well-being *Individual behavior:* Types of PA, sedentary behaviors *Social/cultural:* Modeling/support for PA, social norms and cultural values, institutions, mass media *Environment/ Policy:* Neighborhood, infrastructure, urban planning, health care, policies	(Possible) determinants	Unidirectional influence of the respective components on PA; Change of components over the life course
Levy-Storms et al. (2018) [38]	Systematic review on needs of older adults regarding open spaces, parks, and PA in comparison to younger adults	Biopsychosocial model of health	*Biological/physical needs:* Self-reported physical health, stress, good accessibility, places to rest, ergonomic features *Psychological needs:* Choice, feelings of safety *Social needs:* Foster engagement in social activities, social support, space for social interaction	(Possible) determinants	Reference to interrelationships between components; Inclusion of the environment and the life course
Sallis et al. (2006) [39]	Proposition of a multilevel model of active living that can inform interventions for changes in activity behavior	Ecological model	*Intrapersonal:* Demographics, biological, psychological, family situation *Perceived environment:* Safety, attractiveness, comfort, crime, convenience, accessibility *Behavior settings:* Neighborhood, recreation, home, transport, workplace, school *Policy:* Health care, transport policies, school policies, traffic regulations, neighborhood development policies, media regulations	(Possible) determinants	Unidirectional influence of respective components on PA
Stubbs et al. (2015) [40]	Systematic review on factors that influence participation in PA in adult patients with gon- or coxarthrosis	Socio-ecological model	*Demographic:* Age, ethnicity, sex, BMI *Biological:* Symptoms, pain, aerobic capacity, strength, obesity, stiffness, comorbidities, cardiovascular fitness *Behavioral and skill:* Limb function/balance, gait speed, daily living function *Psychological/cognitive/emotional:* Confidence, quality of life, depression, intention to engage in PA *Social/cultural:* Spouse, employment, exercise in group, social and work functioning *Physical environment:* Outside temperature, rain	(Possible) determinants	Unidirectional influence of respective components on PA

## Results

### Literature Identification

The database search produced a total of 654 results. Three additional records were identified through screening reference sections of eligible articles. After removing duplicates, 477 articles remained. The title screening excluded 351 articles and the abstract screening excluded 85 articles that did not meet the inclusion criteria. Cohen’s kappa was 0.82 for the title screening, and 0.81 for the abstract screening, suggesting a strong level of agreement [41].

For the remaining 41 articles, a full-text screening was conducted, excluding a total of 28 articles with justification (see Fig. 1). A total of 13 articles were included in the scoping review; with six being empirical papers and seven non-empirical papers (e.g., theoretical or conceptual articles) or systematic reviews (see Table 1). All of them were published in English.

### Theoretical Models or Approaches

Concerning the underlying model, a total of eight articles pursued a purely biopsychosocial approach in the tradition of Engel’s model, another five articles employed a socio-ecological approach (see Table 1). In line with our inclusion and exclusion criteria, we included articles based on a socio-ecological model if these models depicted at least a biological, psychological, and social component. The term “transdisciplinary” was used only in some articles that referred to the biopsychosocial model. In contrast, all articles that theoretically framed their work from a socio-ecological perspective included the term “transdisciplinary.”

Most of the theoretical models refer to factors that influence behavior (*n* = 8), health (*n* = 3), or successful aging (*n* = 2). In empirical articles, four articles explicitly employed a biopsychosocial model [25, 27, 29, 32] and two a socio-ecological framework that comprised biological, psychological, and social factors [26, 28]. In non-empirical articles, the biopsychosocial model was referred to in four articles [33, 34, 36, 38], the socio-ecological model in three articles [37, 39, 40].

In empirical papers, a biopsychosocial or socio-ecological approach was employed to inform selection of correlates or assessed effects of PA behavior. Often, such a model also informed statistical modeling procedures, particularly with regard to contextual variables, moderator, and mediator effects. The original authors’ justifications for using a biopsychosocial approach were quite similar to the justifications for using a socio-ecological model. In both cases, authors emphasized the comprehensive and interdisciplinary nature of such models, and their potential to simultaneously examine relationships between biological, psychological, and social factors [26–29, 32]. When using a socio-ecological approach, the original authors also highlighted the complex and dynamic nature of PA behavior [26, 28], a point that was not mentioned in articles based on a biopsychosocial model.

In non-empirical papers, particularly in (systematic) reviews, a biopsychosocial model or a socio-ecological model was used to synthesize and interpret the findings of original research [34, 38, 40]. Authors of review articles underlined that PA behavior is often influenced across multiple levels and that correlates at each level often overlap and interact with each other. Thus, for synthesizing original research findings on correlates of PA behavior, authors chose a biopsychosocial or socio-ecological framework to facilitate a more comprehensive and meaningful interpretation of the data of original studies. However, most original research was conducted from a unidimensional perspective. Thus, the reviews of Kanavaki et al. [34], Levy-Storms et al. [38], and Stubbs et al. [40] cannot answer questions about the interactions of factors at a biological, psychological, and social level. Collins et al. [33] described a multidimensional approach for lifelong sport participation and PA and used a biopsychosocial approach since it provides an effective basis for modeling and manipulating complex human behavior. The multidimensional nature of the biopsychosocial perspective was also emphasized by Kanning and Schlicht [36] in their description of a biopsychosocial model for successful aging. King and King [37] and Sallis et al. [39] employed a socio-ecological approach in their work to inform interventions, scientific research, and politics with regard to the promotion of a healthy lifestyle and active living.

#### Integration of Physical Activity into the Model

Concerning the integration of PA into the models, the majority of included papers (*n* = 9) focused on possible determinants or correlates of PA behavior, i.e., how the different components of a biopsychosocial or socio-ecological model affect PA. Two papers focused on how PA behavior affects the various components of a biopsychosocial model [29, 36]; two articles analyzed both possible determinants of PA behavior and the effect of PA behavior on biopsychosocial indicators of health [27, 32]. Whereas the integration of PA into the biopsychosocial model was not uniform (i.e., PA was either included as the outcome variable, the independent variable, or both), all five articles grounded in a socio-ecological approach examined correlates of PA behavior.

#### Variables Included in the Theoretical Models or Frameworks

The empirical and non-empirical articles did not differ with regard to the included variables in the theoretical models. Thus, in the following, we compare included variables in the models, which were explicitly termed “biopsychosocial,” with those in the socio-ecological models.

The original biopsychosocial model in the tradition of Engel differentiates between biological, psychological, and social aspects. Some of the included articles (see also Table 1) directly used these terms to cluster their examined variables [25, 33, 34, 36, 38], whereas Lämmle et al. [27], and van Roekel et al. [32] group biological, psychological, and social variables into a category system that referred to distal, proximal, and behavioral factors and health indicators. Meisner et al. [29] employ the biopsychosocial model of successful aging by Rowe and Kahn [31] and refer to the three components of successful aging rather than to biological, psychological, and social components.

When examining the variables that were subsumed under each category, the diversity of biopsychosocial approaches in the context of PA research became apparent. Depending on the article’s objective, the original study author(s) grouped a great variety of variables under the respective category. Examined biological variables ranged from BMI to body fat, blood pressure, sex, presence of comorbidities, physical fitness to ergonomic features of outdoor park equipment. Examined psychological variables included perceived stress, motivation, psychopathological problems, cognitive capacity, psychological development, emotions, beliefs about PA, and feelings of safety. Social factors included marital status, employment status, socioeconomic status, PA of relatives and peers, social engagement, role and social functioning, life transitions, social support, stereotypes, behavior settings, and space for social interaction. Age was either assigned to the biological category or to the social category or was considered a covariate (as was also sometimes the case with sex).

Socio-ecological models assessed a similar variety of biological, psychological, and social variables; in addition, socio-ecological approaches placed a greater focus on behavioral (such as screen time, sports team participation, sedentary behaviors, or gait speed and limb function) and environmental aspects (such as neighborhood safety, walkability, outside temperature, and access to facilities). King and King [37] and Sallis et al. [39] included policy as a further component referring to urban planning, health care policies, traffic regulations, media regulations, transport policies, and school policies.

Three empirical articles referred to relationships or interactions between the individual components of the employed model. McNeill et al. [28] included unidirectional relationships between selected components of a socio-ecological model. Interactions between components were integrated into the biopsychosocial model in the articles of Lämmle et al. [27], and van Roekel et al. [32]. The systematic reviews of Kanavaki et al. [34], and Levy-Storms et al. [38] mentioned that interrelationships between various biopsychosocial correlates of engagement in PA behavior can be expected and should be considered in future research. The non-empirical papers of Collins et al. [33], and King and King [37] mentioned a possible change or adjustment of the components over the life course.

## Discussion

The overall aim of the scoping review was to examine how the “biopsychosocial” has been conceptualized in the existing literature on PA behavior. Overall, we only found a limited number of studies that analyzed PA behavior from a biopsychosocial perspective. Notably, only six of the included 13 articles were empirical studies concerned with PA behavior.

### Underlying Theoretical Models or Frameworks

Broadly speaking, the models in the original articles either depicted correlates of PA behavior, or the influence of PA on various biopsychosocial outcomes such as health, quality of life, or successful aging. Several justifications for using a biopsychosocial model or a similar theoretical approach (i.e., transdisciplinary approaches or social-ecological models), which also focuses on biological, psychological, and social aspects, were found in the original articles. Nearly all articles pointed out that a biopsychosocial perspective is fitting to simultaneously examine relationships between biological, psychological, and social dimensions with regard to correlates and effects of PA behavior. Thereby, authors of original articles often referred to the biopsychosocial model’s potential to describe a phenomenon from a multidimensional perspective that takes interactions of various factors into account.

A total of five included articles employed the socio-ecological model in the tradition of Bronfenbrenner [42]. Even though socio-ecological models are models in their own right, we included these articles in the present review as long as it was apparent that biological, psychological, and social factors were part of such a framework; thereby, this work implicitly followed a biopsychosocial perspective. Socio-ecological models in the included articles were concerned with explaining PA behavior or describing a wide range of factors that potentially influence PA behavior in specific populations [43]. Thus, socio-ecological frameworks are used to model not only behavioral influences within and between individuals but also environmental and political influences. Within-person influences often include the biological and psychological components whereas the social and cultural components are summarized under influences between persons or as environmental influences [43].

In contrast to socio-ecological approaches, articles that referred to the biopsychosocial model in the tradition of Engel were not only concerned with influences on PA behavior but also focused on possible biopsychosocial effects of PA behavior; thus extending the scope of the socio-ecological model through considering PA behavior either as the outcome variable or the independent variable.

Taken together, even though both models are theoretical frameworks in their own right, they share a transdisciplinary perspective on PA behavior. When looking at the broader field of health and illness research, there have even been attempts to combine Engel’s biopsychosocial model with Bronfenbrenner’s ecological model in order to better explain health- and illness-related changes in a person [44]. The present scoping review demonstrates the various ways that PA behavior can be included either in a biopsychosocial model or a socio-ecological framework, with neither one offering a concrete basis for the inclusion of PA.

### Variables Included in the Biopsychosocial and Socio-ecological Models

The models that were used in the reviewed articles differed to a large extent with regard to the integrated components and the assessed variables. While some articles followed the original classification into biological, psychological, and social aspects, other articles used different categories such as proximal, distal, interpersonal, intrapersonal, behavioral, environmental, and cultural aspects. These different overarching categories directly go back to the nature of the included models. Whereas most articles that were based on the biopsychosocial model in the tradition of Engel employed the traditional categorization into biological, psychological, and social aspects, socio-ecological models directed attention also to environmental and policy features. Since socio-ecological models in included articles were mainly adopted for studying PA behavior in specific places and populations, a greater focus on the characteristics of places that hinder or facilitate PA behavior can be observed.

Overall, a broad variety of variables was included in the theoretical frameworks. The reasons for such a variety might be traced back to the objectives of the included articles and to the study samples. For example, different aspects may be of significance or of greater significance for the prediction of PA in children and adolescents [26] than for the effects of PA on the quality of life of former colorectal cancer patients [32]. Even though this observed variety of variables makes a clear comparison of approaches nearly impossible, it also demonstrates the potential of a biopsychosocial perspective to be used in a variety of research contexts and to inform transdisciplinary PA research.

The systematic integration of relationships between the biological, psychological, and social components is emphasized in the original work of Engel [7, 35]. However, only few of the included articles empirically analyzed interactions between the individual components [27, 28, 32]. Complex statistical analyses such as path analyses and bivariate correlations [27], moderation and mediation analyses [27, 28], structural equation modeling with a focus on testing theoretical relationships between latent constructs [28], and multivariable linear regression models with subgroup analyses [32] were performed to analyze such complex interactions of model components.

All in all, the aforementioned points illustrate the challenges that arise when applying a biopsychosocial perspective on complex phenomena such as health and/or PA. These challenges become even more complex when differences between individuals and within individuals over time are taken into account.

### Implications for Future Research

The present scoping review shows the benefits of a biopsychosocial perspective on PA behavior. Both the empirical and non-empirical articles in this scoping review advocate for a multidimensional and complex perspective in research on the biopsychosocial correlates and effects of PA behavior. Due to the complex nature of PA behavior, research on correlates and effects of PA should be informed by theoretical approaches that aim to capture this complexity, either with regard to the interactions of multiple correlates of PA behavior or with regard to the complex and interrelated effects of PA.

Even though it seems to be common sense that PA behavior is complex and influenced by a myriad of factors, most empirical evidence is still not based on comprehensive approaches. This point is also demonstrated by the relatively small number of empirical studies identified in this review that explicitly follow a transdisciplinary perspective when examining the correlates of PA behavior.

The included empirical research based on a biopsychosocial perspective convincingly demonstrates that no one factor explains PA behavior; rather it is an interaction of factors at different levels. Grounding empirical research on sound theoretical frameworks, such as the biopsychosocial model or the socio-ecological model, facilitates the identification of important contextual factors and potential confounding and moderating effects when examining influences on PA behavior. However, one major drawback of the models that we analyzed in our scoping review was their missing focus on the complex interactions between the various factors. It is precisely these interactions that add great value compared to a unidimensional view when examining such a complex, multidimensional, and dynamic phenomenon as PA behavior. In future research, approaches are needed that aim to capture these interaction effects.

Thus, for future transdisciplinary PA research, it is a reasonable approach to examine multilevel influences on PA behavior. Such an approach to PA behavior necessitates application of complex statistical procedures (such as growth curve modeling, structural equation modeling, latent class analysis, etc.) that allow identification of intra- and inter-individual and contextual differences as well as dynamic interactions between different factors. Additionally, a multilevel perspective also requires long-term transdisciplinary work from different scientific disciplines such as sports medicine, epigenetics, health psychology, sport sociology, and public health, employing different methodological approaches to capture the full complexity of the underlying phenomenon (i.e., PA behavior). Through considering factors at different levels and particularly their interactions, which necessarily makes such research more complex, it becomes possible to better understand influences on PA behavior. Knowledge gained through such a transdisciplinary perspective will help to develop and implement PA promotion programs, public health strategies, interventions, and policies that more effectively target specific contributing factors for PA behavior in at-risk groups for low PA levels.

Transdisciplinary approaches are also required for recognizing and understanding the effects of PA behavior on an individual and societal level. Asking solely for effects on one level (such as the physiological level) might overlook or even disregard important benefits of PA on other levels (such as the psychological or social) that might also affect health and quality of life, or the outcomes of rehabilitation programs or treatment strategies. Research on the effectiveness of health-related behavioral interventions might also be improved if it considers the biological, psychological, and social benefits of PA.

While transdisciplinary approaches in PA research are more common in some disciplines (e.g., sports psychology and sports medicine or social-psychology), the collaboration between others is still missing. One such transdisciplinary approach might be between the evolving field of epigenetics and sociology and psychology. Outside the field of sport science, researchers have already begun to argue that epigenetics should expand its scope beyond molecular biology research only [45], and rather complement its research agenda with sociological and psychological models, which might help capture environmental influences on gene expression [2]. By including epigenetics as a sub-aspect of the biological component, future transdisciplinary PA research could generate new insights into the genesis of many health-related phenomena through a focus on interactions between genetics and PA [46], or the influence of the environment on gene expression in the context of adaptations to training, exercise, and PA [47].

### Limitations of the Scoping Review

Some important limitations of our review need to be considered. The first limitation lies in the small number of included studies. The relatively strict inclusion and exclusion criteria could be a reason for this. However, they were necessary to limit the scope of the review. We acknowledge that further insights could be gained by broadening the search strategy, i.e., also including the areas of exercise and sports.

Additionally, data may be incomplete because some studies might have been published in another language or indexed in other databases. However, through screening of reference sections of eligible studies, we aimed to lower the risk of bias across studies.

Since we solely included the terms biopsychosocial and transdisciplinary in our search, articles that only implicitly employ such a perspective and do not use either of the terms were not included in this review. As a consequence, often cited papers such as the articles from Bauman et al. [48], or Bryan et al. [46] have not been identified in our search. Bauman et al. [48], for example, examined correlates and determinants of PA with the goal to develop a multi-level ecological model for understanding the causes of PA behavior. The authors aimed to develop a model that also takes into account how etiological factors differ between PA domains, areas of life in which activity is observable, and country, age, sex, ethnic origin, and socioeconomic status. Bauman et al. [48] concluded that individual-level factors such as age, sex, health status, self-efficacy, and previous PA are consistent correlates of PA, but the physical and social environments, such as policy, economic conditions, societal norms, urbanization, industrialization, or interpersonal relations are also important determinants of PA behavior. Not least, they also argued that a genetic and evolutionary physiological component has to be considered when analyzing the correlates of PA behavior. Against this background, the model of Bauman et al. [48] can clearly be considered a biopsychosocial model despite the fact that they did not mention this term in their paper.

Similarly, the transdisciplinary model of PA behavior by Bryan et al. [46] also offers an exemplary approach for a multi-perspective analysis of PA. Bryan et al. [46] modeled the complex interactions of different components for behavioral change using the example of an intervention that promotes engagement in PA. In this regard, they explicitly considered the individuality of people and thereby individual differences as a reason to employ a transdisciplinary approach. They not only included a physiological, a genetic, a psychological, and a behavioral component into their theoretical model but also considered bidirectional relationships between components in order to explain (possible) effects and determinants of PA. However, this is a fundamental difference to the model of Bauman et al. [48], they completely abandoned the social component in their transdisciplinary approach. Although the article is generally very informative and helpful for designing intervention programs, the lack of a social component is a relevant shortcoming, as PA behavior is highly influenced by social and environmental factors.

## Conclusion

This scoping review provides a first overview of biopsychosocial models in the context of transdisciplinary PA research. In principle, biopsychosocial models refer to PA behavior from a holistic perspective including biological, psychological, and social aspects and beyond such as the wider environment or politics. Therewith, in principle, a biopsychosocial approach yields great potential for an exhaustive, transdisciplinary perspective on PA behavior. The findings of the present scoping review illustrate that socio-ecological models mainly focus on the determinants or correlates of PA behavior, whereas biopsychosocial models in the tradition of Engel adopt a broader perspective referring both to the determinants and to the effects of PA behavior. Additionally, biopsychosocial models mostly focus on promoting health through PA.

The included articles illustrate that the adoption of a biopsychosocial perspective may assist to capture and understand the complex phenomenon of PA behavior. One of the main advantages of a biopsychosocial perspective in health research is that it allows consideration of individuality and individual differences on different levels. Thus, the observed heterogeneity with regard to the employed models and with regard to the assessed variables at the biological, psychological, and social levels can be considered a strength of the biopsychosocial perspective. Individuals of different ages with different health status and different performance capabilities are likely to display inter-individual variation with regard to the determinants and effects of PA. It is also likely that the determinants and effects of PA behavior change over the life course of an individual, thus displaying intra-individual variation. Consequently, inter- and intra-individual differences at a biological, psychological, and social level are likely to play an important role in maintaining an active lifestyle as well as in individual training responses, or in predicting improvements in athletic performance [2, 33].

Taken together, the included articles may give a first idea on how to holistically conceptualize PA, which might be of help when designing programs for the promotion of PA. In this sense, the present scoping review not only shows the potential of looking at PA behavior from a biopsychosocial perspective but also highlights the need for further transdisciplinary research in this area.

## Data Availability

Data analyzed during this scoping review are included in this published article (in the tables summarizing the included studies). Reasons for the exclusion of studies can be requested from the corresponding author.
